# Development and validation of a novel suspended particulate matter sampling device for analysis of particle-bound microbial communities

**DOI:** 10.1099/mic.0.001538

**Published:** 2025-03-06

**Authors:** Fuad J. Shatara, Kiyoko Yokota, Justin Peschman, Azul J. Kothari, Jacob Schoville, Liyuan Hou, R. Preston Withington IV, Cole F. Beale, Maria Pelusi, Kyle M. Boldon, Jennifer Withington, R.P. Withington III, Hannah Nicklay, Michael R. Twiss, Charles J. Paradis, Erica L.-W. Majumder

**Affiliations:** 1 Department of Bacteriology, University of Wisconsin-Madison, 1550 Linden Drive, Microbial sciences Building 5545, Madison, WI 53706, USA; 2 Department of Biology, State University of New York at Oneonta, 108 Ravine Parkway, Oneonta, NY 13820, USA; 3 Department of Geosciences, University of Wisconsin-Milwaukee, 3209 N Maryland Ave, Lapham Hall 348, Milwaukee, WI 53211, USA; 4 Department of Environmental Sustainability, Health and Safety, Rochester Institute of Technology, 1 Lomb Memorial Drive, Rochester, NY 14623, USA; 5 Department of Biology, Clarkson University, 8 Clarkson Avenue, Potsdam, NY 13676, USA; 6 Lake Superior National Estuarine Research Reserve, University of Wisconsin-Madison, Division of Extension, 14 Marina Drive, Superior, WI 54880, USA

**Keywords:** freshwater, methodology, microbial community, microplastics, sampling, size-fractionated recovery, suspended particulate matter

## Abstract

Biotic and abiotic materials attachment to suspended particulate matter in aquatic systems can increase their toxicity and health impacts and has led to an increased need for consistent sampling across various compartments. Sedimentation traps and continuous flow centrifuges are the traditional tools for sampling suspended particulate matter, while manta trawls have been widely used for surface water sampling of suspended or floating microplastics. Limitations, however, exist in the cost of sampling and infrastructure needed to deploy such devices. Here we report on the construction and usage of a novel suspended particulate matter sampling device, the microParticle Obtaining Trap (mPOT). Quality control testing of the mPOT showed suspended particle recovery rates of >90% for particles 100 µm and larger, while field sampling of groundwater, lake and river water shows consistent, size-fractionated recovery of particulate matter. The mPOT is well suited to sample systems not easily accessible by boat or for particles not commonly recovered by common suspended particulate matter sampling and for collection of particles smaller than 300 µm in size.

**Impact Statement**

Suspended particulate matter and its impact on aquatic ecosystem health are largely influenced by anthropogenic runoff, which can carry chemical contaminants and microbes of emerging concern long distances across water bodies. By capturing suspended particulate matter, we can gain a better understanding of what biotic and abiotic materials are being transported along their surface and generate targeted strategies within our water infrastructure to improve both our clean water and sanitation practices. Additionally, the simple and inexpensive design of the microParticle Obtaining Trap will allow for citizen scientists to collect samples from bodies of water of interest to their community to analyse for microplastic pollutants, addressing a rising global concern.

## Data Availability

Sequencing data supporting this publication are openly available at BioProject accession: PRJNA1177843, ‘Development and validation of a novel suspended particulate matter sampling device for analysis of particle-bound microbial communities.’

From the Genesee River, samples are listed under accession numbers SAMN4455333 (Site A Bulk), SAMN44455334 (Site B Bulk), SAMN44455335 (Site C Bulk), SAMN44455336 (Site A Particulates), SAMN44455337 (Site B Particulates) and SAMN44455338 (Site C Particulates).

From the Moses-Saunders Power Dam, samples are listed under accession numbers SAMN44455339 (Bulk water) and SAMN44455340 (Particulates).

From Barker’s Island, samples are listed under accession numbers SAMN44455341 (August Bulk Water), SAMN44455342 (August Filtered Water), SAMN44455343 (Bloom Water), SAMN44455344 (August 100 µm Particulates), SAMN44455345 (August 300 µm Particulates), SAMN44455346 (August 500 µm Particulates), SAMN44455347 (October 100 µm Particulates), SAMN44455348 (October 300 µm Particulates), SAMN44455349 (October 500 µm Particulates) and SAMN44455350 (October 1,526 µm Particulates).

## Introduction

Aquatic suspended particulate matter (SPM), both organic and inorganic, originates from a wide variety of point- and non-point sources [1] and often undergoes additional biogeochemical transformations before accumulating in rivers, streams, groundwater and oceans [2]. In the environment, biotic and abiotic materials attachment to SPM may influence their toxicity and health impacts [3,4]. SPM-associated microbiota often results in distinct community compositions compared to those in the bulk water [5,7]. As such, there is a need to sample and recover SPM in various aquatic systems reliably to accurately characterize their potential health impacts and bound microbial communities within the environment [8,10].

The recovery of SPM via environmental sampling has taken a variety of forms and commonly relies on devices such as continuous flow centrifuges or sedimentation traps [11]. Passive samplers, such as sedimentation traps, can allow for time-integrated recovery of material, while active samplers allow for snapshot sample collection. Passive samplers are commonly used for their low maintenance requirements and flexibility in sampling locations for deployment, while the active samplers tend to have higher maintenance requirements and can only be deployed in locations with proper infrastructure for installment, including power sources and systems to mount the devices [10]. Appropriate selection of sampling devices largely depends on the specific study system and the research objectives, with passive sampling being employed for time-integrated sample collection and active sampling being used to collect samples that are sensitive to transformation over time.

Microplastics, a common component of SPM, characterized by sizes <5 mm, have been detected in all oceans and major bodies of water around the world [12,13]. The ubiquity of environmental microplastics drives the global research community to study the influence of these particles in their respective environments, with a large focus on human and animal health impacts [14,15]. In microplastic studies, these sampling endeavours rely upon conducting surveys with trawl nets, commonly manta or neuston nets [16]. These nets have mesh sizes of 330–335 µm and sample within the top 100 cm of the surface water while being trawled behind a boat and sampling large volumes of water, with typical deployments lasting up to 30 min [16,18]. Usage of these nets tends to underreport microplastic concentrations, especially those within the <300 µm range. This size category includes microplastic fibres (e.g. clothing), fragments (e.g. paint) and particles (e.g. tyre dust), all prevalent in the aquatic environment [19]. However, trawl nets have been found to overestimate the concentration of buoyant particles [20] and introduce additional microplastic contaminants from the nets themselves, which are often made of nylon. Sampling with nets also becomes difficult during adverse weather conditions (high wind and waves, which can also lead to increased sediment resuspension and net clogging). Clogging of nets can also be a major sampling issue in locations with high neuston biomass, where nets with meshes smaller than 330 µm often become clogged and halt the sampling [21,22].

While sampling SPM in aquatic systems primarily utilizes a variety of instruments, there is a lack of standardized or inexpensive methods that can be deployed without specific infrastructure, intensive monitoring, and that can adapt to the aims of different studies. Better characterization of the size, vertical distribution and attached biotic and abiotic materials of the SPM will augment our ability to design and model studies to gain a more holistic understanding of the impacts of SPM across different water body types.

Recognizing the need for inexpensive and consistent recovery of SPM, we have constructed and validated a novel sampling device. The device, called the microParticle Obtaining Trap (mPOT), comprises a series of stainless-steel mesh screens housed within a stainless-steel stock pot. The mPOT was designed with the objective of standardizing sampling practices and ensuring consistent recovery of material through an easily modifiable and comparatively inexpensive design with parts readily available to the public. Modifications to the external housing allow for sampling in different environments, including applications for passive or active sampling, depending on the desired water source. Modifications to the internal mesh screens, both in number of screens and mesh size, allow for size-fractionated recovery of these particles. The redundancy of the filters within the mPOT allows for usage in both the recovery of particulate matter from filtered water, as well as the ability to filter sediment similar to common sieving strategies. Additionally, the comparatively low price and ease of parts sourcing for the mPOT allows for citizen science monitoring. In this report, we describe the construction of our sampling device and the validation of the mPOT by lab-based studies, as well as deploying it on a groundwater well pump, hydroelectric dam, riverbank and estuarine bay of Lake Superior. Through the usage of mPOT, we were able to identify distinct microbial communities forming on SPM when compared to freshwater, and changes to microbial community populations between different sizes of SPM.

## Methods

### Prototype and mPOT device description and parts required

**Fig. 1.**
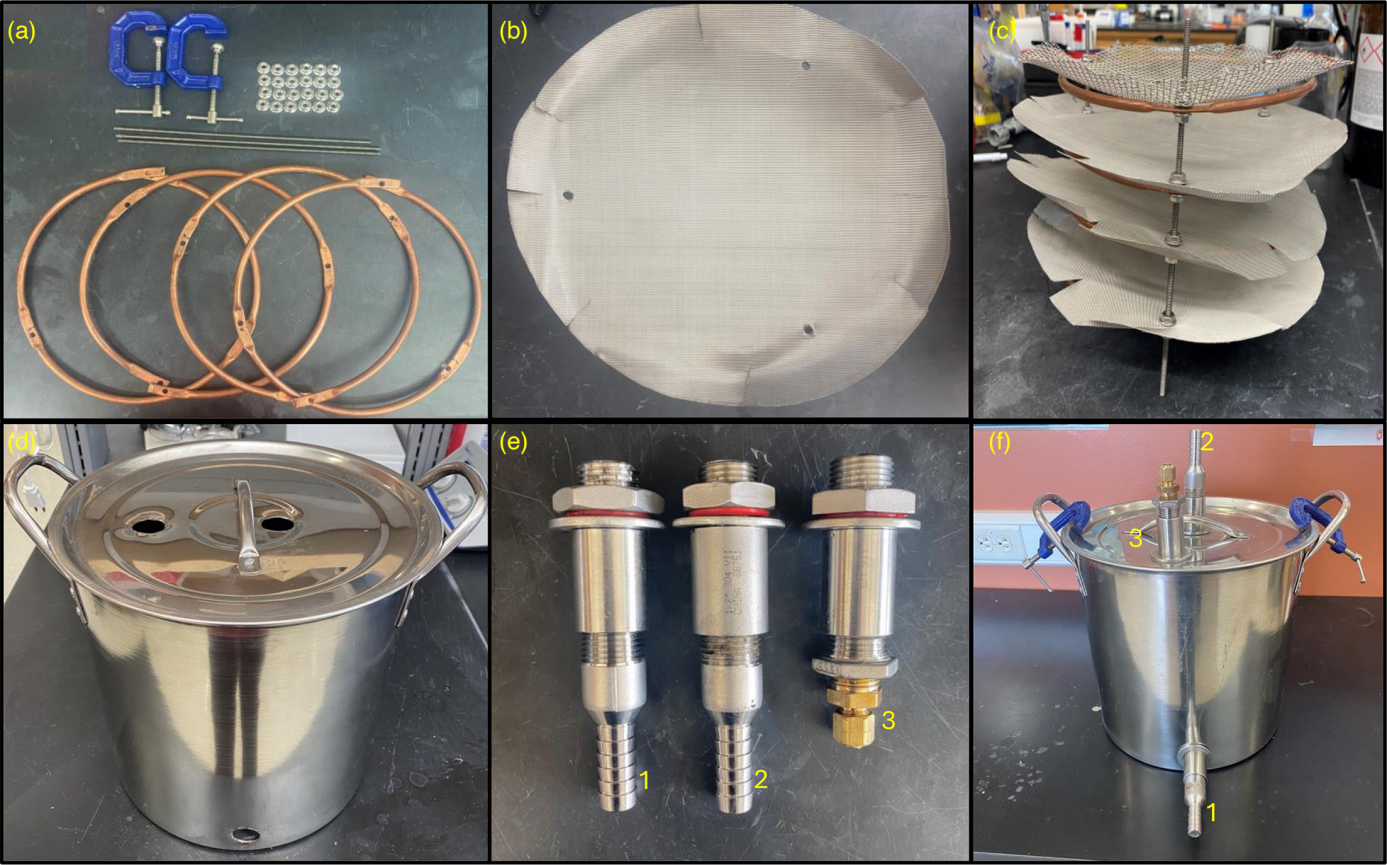
Parts and construction of an mPOT. (**a**) Copper rings, rods and flange nuts for construction of filtration portion and C-clamps for sealing device. (**b**) Cut stainless-steel mesh filters with holes for scaffold rod insertion. (**c**) Assembled filtration portion of mPOT, 1,526 µm filter on top, followed by 500, 300 and 100 µm filters. (**d**) Housing unit and inflow and outflow drill hole locations. (**e**) Bulkhead and hose barb fittings for inflow [1], outflow [2] and gas exchange [3] for housing unit. (**f**) Assembled housing unit.

Construction of the prototype device and mPOT entails two separate pieces assembled independently (Table 1, Fig. 1). The filtering portion, identical between the prototype and the mPOT, comprises a series of stainless-steel screens of 1,526, 500, 300 and 100 µm, respectively (Arbour Fabricating #12×12/100, #12×12/300, #12×12/500, #12×12/1526) in tandem with one another, held in place by three stainless steel #8–32 threaded rods (Grainger#10W721) and concentric rings of copper tubing (Grainger#3P669). Filter sizes were selected in order to limit the potential for clogging within the mPOT, as well as to recover particles <300 µm, which are not commonly captured with trawls and can significantly increase total recovered SPM [23]. The housing portion of the prototype consisted of a 5-gallon homer bucket (Home Depot #131227), while the mPOT consists of a stainless-steel stock pot (Amazon #B0018KLOCO). In both designs, holes are drilled in the lid and near the base insert fittings to serve as inflow and outflow points for sample water to pass through. The fittings also limit contamination into the filtering portion from atmospheric deposition as well as ensuring all collected water passes through the filters. However, with the prototype design, initial testing as described in section 2.8.1 showed that small orange fibres from the housing unit were caught on the filters, leading to the usage of a full stainless-steel device in the final mPOT design. In total, the cost per mPOT device was approximately $171.58 (Table 1), which is a fraction of the cost of most manta nets that can range in price from $500 to $3,000 depending on size and material composition.

**Table 1.** Parts and costs (in U.S. Dollars) associated with the construction of the mPOT. The vendors listed are those used in this study; other vendors are available that provide these materials at similar costs

Part	Vendor	Catalogue no.	Cost	Quantity per device	Cost per device
Steel pot	Amazon	B0018KLOCO	$21	1	$21
Mesh filters	ArborFabricating	Varies	$15	4	$60
Copper tubes (15.2 m)	Grainger	3P669	$32	8 ft (makes 6)	$5.33
Stainless steel #8–32 threaded rods (1 m)	Grainger	10W721	$3	2 ft (makes 1.33)	$2.25
Flange nuts, #8–32 (50 pk)	Grainger	41MG58	$6	24 (Makes 2)	$3
½-inch MNPT × ½-inch hose barb fitting for inlet and outlet	Grainger	3LZ83	$15	2	$30
½-inch MNPT × 3/8-inch FNPT bushing for P-trap	Grainger	6JM34	$3	1	$3
½-inch stainless steel bulkhead fitting, female	Homebrewing	P3336	$13	3	$39
Sioux Chief ¼-inch compression × 3/8-inch MIP lead-free brass adapter	Menards	6801546	$2	1	$2
IRWIN 1-½-inch C-clamp	Menards	2442050	$3	2	$6

### Construction of the filtering apparatus (mPOT)

Construction of the filtering apparatus required the coiling of copper tubing into a ring the size of the housing unit’s internal diameter. At three points along the copper tubing, the ring was flattened with a bench vice (Rock Island 541B) to allow for a flat surface to drill a 4.76 mm (3/16 inch) hole with a twist drill where grade 316 stainless-steel threaded rods are inserted to create a scaffolding (Fig. 1a). Mesh screens were then cut with tin snips to slightly larger than the diameter of the housing unit and approximately 2.5 cm lines cut towards the filters' centre to ensure that, once inserted, the filters would create a bowl within the interior to limit gaps between the edges of these filters and the interior of the housing unit (Fig. 1b). The steel rods are then cut to a height of 17.8 cm (7 inches) using a band saw (Jet E162686) to serve as a scaffold for the mesh screens and copper rings. The copper ring was then overlaid with a corresponding mesh screen to mark where holes were then drilled in the mesh, ensuring correct alignment when inserting the scaffolding rods.

To assemble the filtration portion, one of the four copper rings was inserted through the three rods and the corresponding mesh screen to lay the screen atop the ring. The ring and mesh were then secured together using flange nuts (Grainger 41MG58), secured tightly against the bottom of the flattened portion of the ring and atop the hole drilled in the mesh screen. This was then repeated for each additional mesh screen, beginning with the smallest mesh filter on the bottom to the largest mesh filter on top (Fig. 1c).

### Construction of the housing unit

Three 25.4 mm (1 inch) diameter holes were drilled into the housing unit using a step drill bit (Irwin IWAS10239DF) to insert the fittings that allow for inflow and outflow of the mPOT, as well as for gas exchange once an mPOT is sealed (Fig. 1d). The inflow and outflow were assembled by attaching the 12.7 mm (½ inch) stainless steel bulkhead fittings (Homebrew P3336) to the hose barb fittings (Grainger 3LZ83), while the gas exchange portion requires the bulkhead fitting to attach to a steel bushing (Grainger 6JM34) followed by a brass compression adapter (Menards 6801546) (Fig. 1e). The inflow and gas exchange were inserted into the holes drilled into the lid of the mPOT, with the inflow being inserted near the centre of the lid. The outflow piece was inserted into the holes drilled into the front of the housing unit near the bottom to ensure that water does not pool in the base of the housing unit (Fig. 1f).

### Assembly of mPOT

Once both portions were constructed, the filtration apparatus was inserted into the housing unit and the lid is secured to the base of the housing unit using two C-clamps on opposite ends of the lid to create a seal (Fig. 1f). Prior to deployment, an mPOT should be autoclaved, and then the barbed hose fittings should be sealed with Parafilm to limit contamination from airborne microplastics. These seals should remain in place until the mPOT is deployed in the sampling location, at which time hoses should be affixed to the inflow and outflow fittings. The specific set-up for each use case is described in the corresponding section.

### Recovery and analysis of particles from filters after sampling

To remove debris from the filters, mPOT must be disassembled. For recovery of biological materials from these filters, this disassembly took place within a biosafety cabinet. The filters were rinsed with sterile water in a spray bottle into an aluminium collection tray to remove and collect larger particles (<500 µm). Filters were then scrubbed with steel wool, followed by bath sonication (Fisher Scientific 15336121), to aid in removal of particles collected on the 300- and 100 µm mesh screens. Material removed from the mesh screens by bath sonication was then filtered through a 1.5 µm glass microfibre filter (MilliporeSigma WHA1827047) for further analysis of SPM. Recovered SPM was then separated for either genomic DNA (gDNA) extraction or microplastic analysis. Separation of the microplastic debris from other particulate matter and debris recovered was conducted using the National Oceanic and Atmospheric Administraton Marine Debris programme method [24]. Briefly, the size-fractioned recovered material from each filter was exposed to 30% hydrogen peroxide solution (ThermoFisher Scientific H3254) to remove organic material from the particulates. Following this, the remaining solid materials were collected and subjected to density separation, which uses a high-density sodium chloride solution (1.2 g/cm^3^) to recover plastics that have a lower density, including some of the most commonly found plastics in aquatic systems, polyethylene and polypropylene [25]. Wet peroxide oxidation and density separation may need to be repeated multiple times to sufficiently remove all organic matter.

Recovered materials are then analysed via attenuated total reflectance Fourier transform infrared spectroscopy (ATR-FTIR, Nicolet iS50R) in transmission mode, with a resolution of 4 cm^−1^, in a scanning range of 400–4,000 cm^−1^, with 30 scans to identify polymer type of the recovered microplastics. Collected chemical spectra of the materials are compared against reference plastics [26]. This allowed for identification of polymers within the material recovered following chemical and gravimetric separation. Following ATR-FTIR analysis, particulate matter was sputter coated in 5 nm of gold using Leica ACE600 and scanning electron microscopy (Zeiss GeminiSEM 450) was used to verify size fractionation of the recovered material.

### Recovery and analysis of microbial communities after sampling

For gDNA extraction, the recovered SPM particles obtained in section 2.5 were filtered onto a 0.22-µm polyethersulfone membrane before extraction, following the protocol in the Qiagen DNeasy PowerSoil Kit (Qiagen 47014). Recovered DNA from this extraction was quantified using Qubit before DNA amplification and sequencing, following the method of Chatman *et al.* [27]. Briefly, extracted DNA was amplified using primers targeting the V4 region 16S rRNA gene, and the amplified genes were then verified using gel electrophoresis before being normalized to 20 µl using the SequalPrep Normalization Kit (Life Technologies). Normalized products were then sequenced on an Illumina MiSeq to determine the composition of the microbial community [28]. Sequencing results were processed in Mothur [29], and visual and statistical analyses were conducted in R [28] to calculate diversity indices [30,34].

### Device performance with standard microplastic beads

To determine the ability of the mPOT to recover SPM in aquatic environments, a device was constructed as described in sections 2.2–2.4, containing four mesh screens of sizes 1,526, 500, 300 and 100 µm. Initial in-lab device performance testing of particle recovery rates was conducted for standard polyethylene and polystyrene microplastic beads of 100 (Cospheric UVPMS-BB-1.13), 300 (UVPMS-BO-1.00), 500 (UVPMS-BR-1.20) and 5,000 (Aldrich #331651) µm in size. 0.1 g of 100, 300 and 500 µm beads and 5 g of 5,000 µm beads were added together to 1.5 l of water in a glass flask. The mixture was then poured into the sampling device via a funnel through the inlet nozzle on the lid of the housing unit. Water was allowed to flow through each of the filters in series, collecting the particles. Filtrate was collected in a glass bottle to check for plastics exiting the mPOT.

Following flow through, the filtering apparatus was disassembled. Microplastics on each screen were rinsed off using a spray bottle of sterile water or scrubbed off with steel wool before being collected in an aluminium weigh boat. The collected material was then vacuum filtered through a 1.5-µm glass microfibre filter to recover all plastic beads. The beads were dried overnight at 50 °C and weighed again the next morning to determine percent recovery by mass.

### Field validation of particle recovery by sampling groundwater from a high-capacity well

**Fig. 2.**
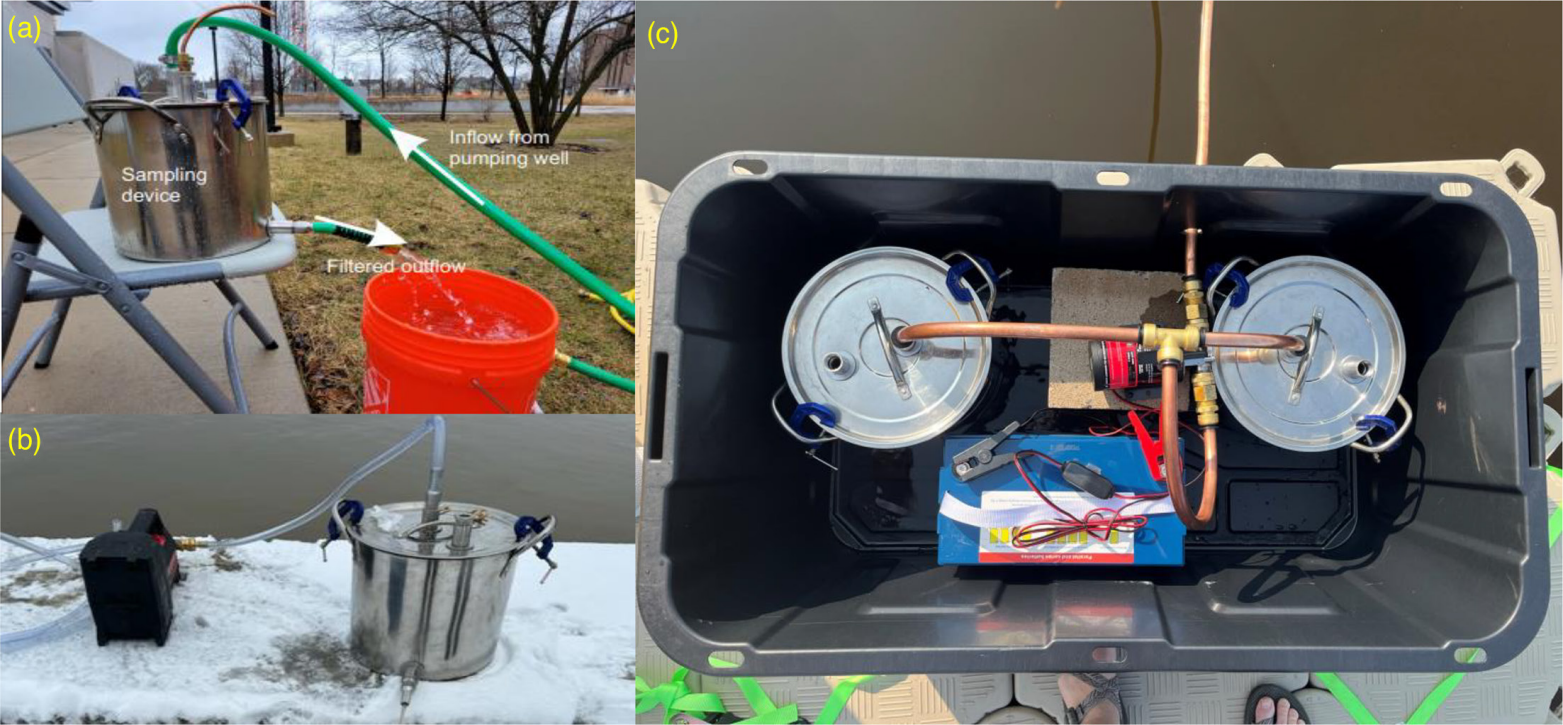
Sampling setup on (a) Niagara Dolomite Aquifer, (**b**) Genesee River, (**c**) Barker’s Island.

One assembled sampling device was sent to the University of Wisconsin-Milwaukee in order to sample water from a deep groundwater high-capacity well completed in Wisconsin’s Niagara Dolomite aquifer (Fig. 2a). This test was aimed at testing the mPOT device in groundwater sampling and detecting potential microplastic pollution from the nearby Deep Tunnel System, which captures storm and wastewater overflow within the Milwaukee region. Any leakage of this system could introduce a variety of pollutants into the aquifer [35,36]. The 6-inch diameter steel pumping well reaches 350 feet below the surface, the same depth and formation as the Deep Tunnel (43.07583 N to 87.88449W) and was pumped through a garden hose into the mPOT at a rate of 350 l/min. In total, over 1,000 l of groundwater pumped from the well station was filtered through the mPOT and the mass of SPM on each of the filters was determined.

### Sampling from the Moses–Saunders Power Dam

Initial testing of the prototype sampling device was conducted on the Moses–Saunders Power Dam in Massena, NY, which spans the St. Lawrence River. The prototype device was installed at the end of a series of autonomous water quality sensors sampling water prior to passing through a turbine (No. 32) located furthest to the south in the dam. Sampling took place during October 2021. The sampling device was left overnight and filtered approximately 1500 l of water before being disconnected and shipped back to the lab in Wisconsin for analysis. Bulk water was also collected from the dam in order to compare the SPM-bound communities and total water communities.

Particulate matter from the screens was obtained through washing and sonicating as described in section 2.5. An aliquot from each screen was analysed with Scanning Electron Microscopy (SEM) and microbial community sequencing. Recovered particulate matter was sputter coated with 5 nm of gold using LeciaEM ACE600 before imaging with a Zeiss GeminiSEM 450 to verify the size of recovered material on each of mPOTs filters.

### Sampling from the Genesee River

Sampling on the Genesee River was conducted by a team of one undergraduate student and two adult volunteers in January 2023. The team was briefed during a virtual meeting on the usage and deployment of an mPOT and the attachment of a cordless transfer pump (Grainger 49V05) for inflow of water. The field sampling was performed at three sites (Site A—latitude: 43.143, longitude: −77.615; Site B—latitude: 43.144, longitude: −77.614; Site C—latitude: 43.147, longitude: −77.610) along the shore of the Genesee River as it passes through Rochester, downstream of a United States Geological Survey monitoring station (USGS Station 04231600). At each site, water was pumped through a different sampling device to compare recovery of particulate matter and water samples between locations. First, 1 l of unfiltered river water was collected in a sterilized glass bottle. Then the outflow tube from the pump was connected to the mPOT inlet (Fig. 2b). Water was pumped through until the battery was depleted. During pumping, 1 l of filtered water was collected in another sterilized glass bottle. Sealed devices and river water in glass bottles were shipped back to the lab in Wisconsin. Particulate matter and genomic DNA were recovered and extracted as described above.

### Sampling at Barker’s Island within the St. Louis River Estuary

Sampling at Barker’s Island within the St. Louis River Estuary aimed at identifying microbial communities attached to size-fractionated particulate matter. Sampling was conducted during two 1-month sampling periods, from July to August and September to October of 2023. Two mPOT devices were set up, and water was pumped (Barracuda 6913319) from the bay via copper tubing and a splitter to be filtered through the devices simultaneously to limit clogging. The pump was connected to an external battery (WEIZE BAC-0089), which was changed out periodically; therefore, sampling was not continuous during the months. The devices were secured inside an industrial storage tote (Menards 6451501), and the tote secured to the docks using ratchet straps (Menards 2352652) (Fig. 2c).

Filtered water was collected from the outflow of the mPOT following sampling as well as directly from the bay at the end of the first sampling period for comparison to the particle-bound community. Water was also collected from Barker’s Island Swimming Beach, where an algal bloom occurred in August of 2023. Following sampling at Barker’s Island, the mPOT devices and water were brought back to Madison, Wisconsin in a cooler on ice before processing. Particulate matter was recovered from each screen of the devices independently. Samples from each size of filter were pooled between the two devices before processing for 16S rRNA gene sequencing of the V4 region as described in section 2.6.

## Results and discussion

### Lab particle recovery validation testing

**Fig. 3.**
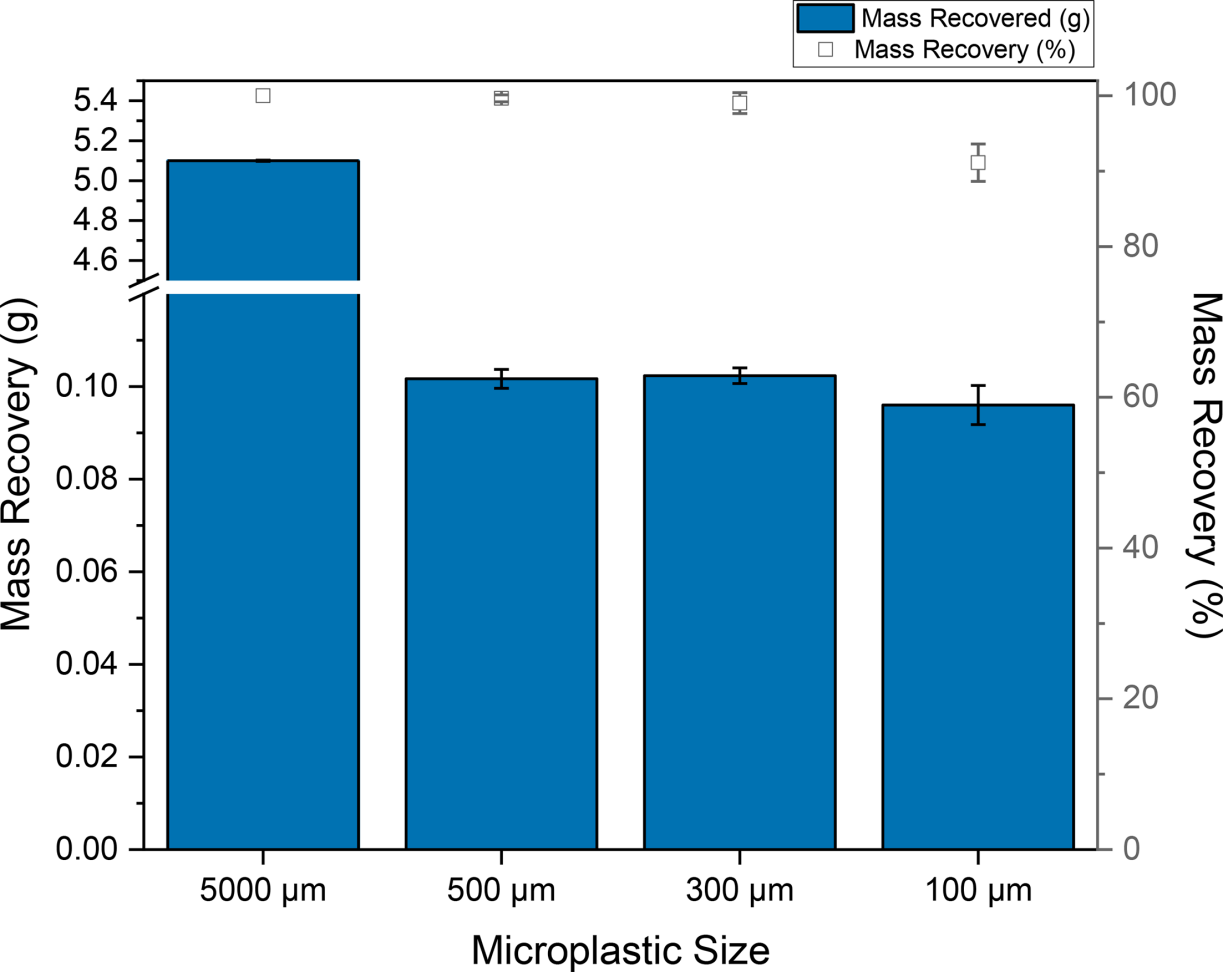
Microplastic recovery, by mass and mass %, from in-lab validation of the mPOT device.

In lab testing of mPOT aimed to quantify the device’s ability to recover standard microplastics particles of varying sizes on each of its screens. In recovering these beads, it was noted that, while most beads were recovered on the mesh size that corresponded to the bead size, some were captured on a mesh either larger or smaller than the particle size. Previous testing by pumping water into the mPOT through nylon tubing showed that some particles adhered to the interior of the tubing without continuous flow of water; therefore, copper tubing was used in its place in future tests. Removal of particles from the mesh screens was noted to be most difficult on screens where particle sizes matched the screen size, lodging into gaps between the stainless steel. For each of the 100, 300 and 500 µm particles, 0.1 g of plastic beads were tested for recovery, while 5 g of beads were used for the 5,000 µm particles. Particle recovery by mass was demonstrated to be 100% for 5,000 µm beads, >99% for both 300 µm and 500 µm and >90% for 100 µm beads (Fig. 3). These results showed the capability of mPOT to recover and separate SPM down to 100 µm in size, a significant improvement over the 300 µm trawl nets, the current standard method for aquatic microplastic sampling.

### Field particle recovery validation testing

**Fig. 4.**
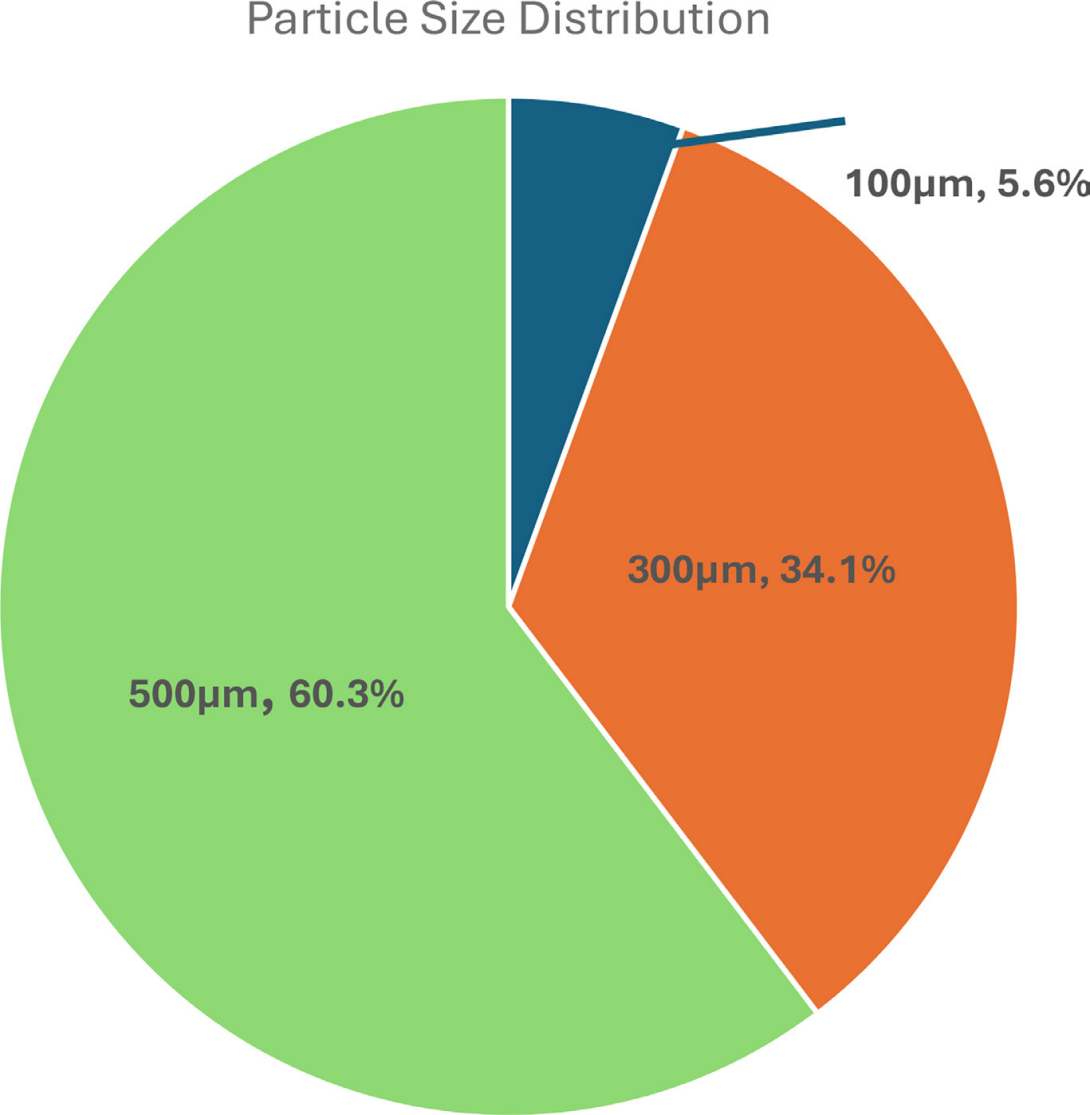
Size fractionated particle recovery rate from Niagara Dolomite Aquifer.

Sampling from the Niagara Dolomite aquifer aimed to test the mPOT's ability to recover SPM from aquatic systems, rather than the standardized particles from the in-lab testing. Annual groundwater samples collected from the pumping well are thought to be of relatively low turbidity and total suspended solids due to their consistently clear appearance upon visual inspection and ease of hand-driven filtering through 0.22-μm syringe filters (Paradis, C.J., personal communication, 2025). Thus, it is possible that sub-0.22 μm solids, natural and/or anthropogenic, are present in the aquifer fluids. From the material covered after pumping a large volume of water, 200 mg was recovered from the filters. 60.3% of the recovered material was adhered to the 500 µm filter, 34.1% to the 300 µm filter and the remaining 5.6% to the 100 µm filter (Fig. 4). No materials were recovered on the largest 1,526 µm filter. Raman spectroscopy was used to analyse recovered particulate matter and identify microplastic pollutant**s**; however, no microplastics were identified. Although potential leakage of stormwater from the Deep Tunnel into the aquifer was not detectable in this study, the recovery of SPM from groundwater by pumping from this well could be used to monitor for leaks in the future.

In this case, particulate matter was recovered from groundwater samples and validated the separation of materials through an mPOT via the average particle distribution analyses. Although no plastics were found, the mPOT allowed for recovery of particulates from previously understudied sources, unreachable by the conventional sampling methods like trawl nets. By adjusting filter sizes and pumping rates, longer-term passive sampling studies with continuous flow could provide additional insight into sources of microplastic contamination in groundwater and other similar groundwater sources.

### Passive sampling in a hydroelectric power dam

**Fig. 5.**
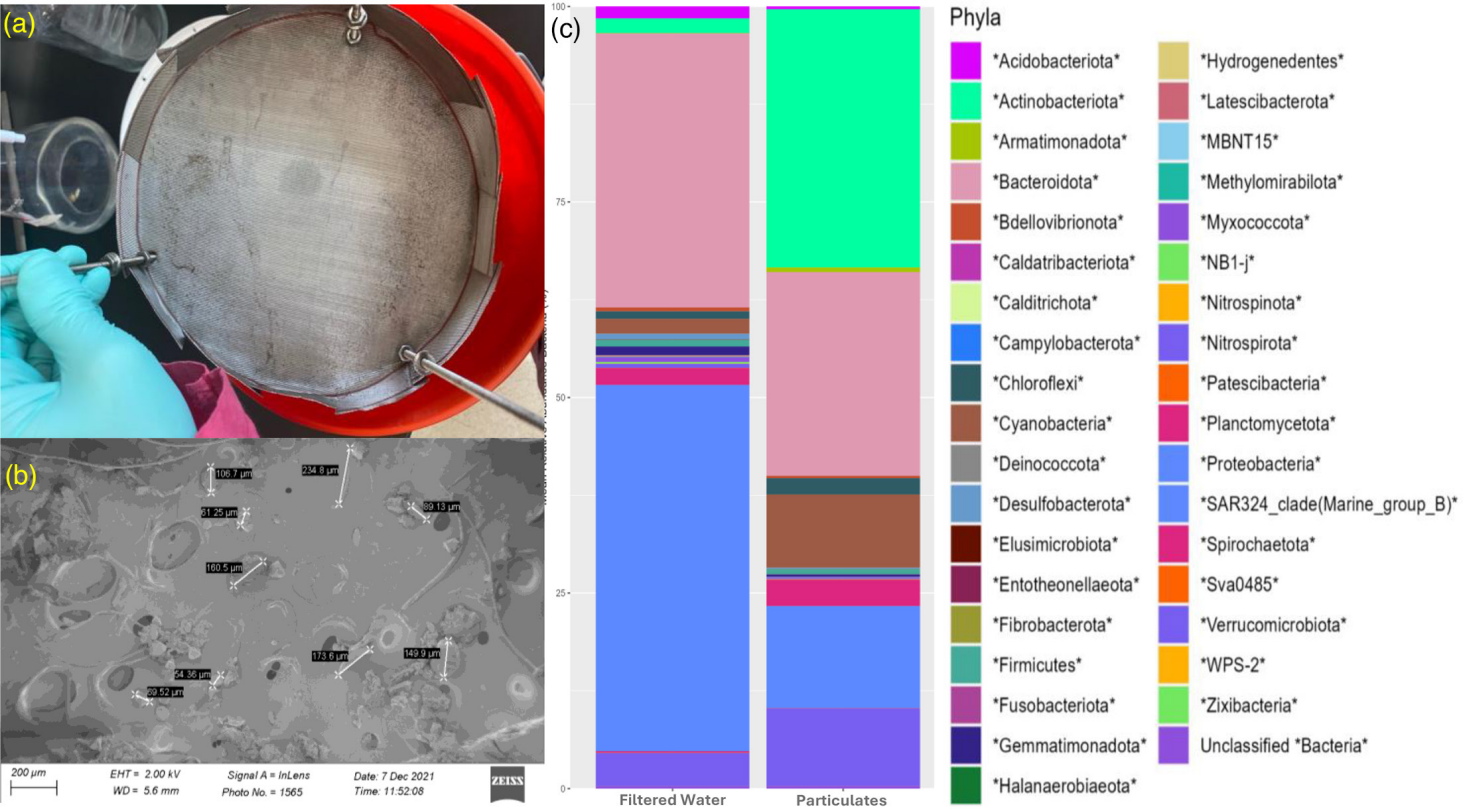
(a) 100-µm mesh screen following 24 h passive sampling using an mPOT installed at the Moses-Saunders Power Dam; (**b**) SEM image of particulate matter from that same 100 µm mesh screen; (**c**) Taxonomic bar plot of bacterial phyla relative abundance.

Pilot testing of the mPOT on the Moses–Saunders Power Dam sought to characterize microbial community populations in homogenized bulk water and SPM from the Saint Lawrence River and identify microplastic pollutants within the SPM (Fig. 5a). In this study, we hypothesized that microbial communities obtained from water are more diverse than communities attached to SPM. Sizes of particulate matter recovered on each screen corresponded to the size of the mesh (Fig. 5b), with the exception of fibrous materials which varied in length across each of the screens. Since fibres are long on one side but narrow on the other, capture of fibres by filters is likely to undercount them, though this validated that mPOT captures fibres of various sizes. However, microplastics were not identified among the recovered particulate matter. DNA was extracted from the biomass bound to the recovered particulate matter from each mesh screen and from water collected from the dam to determine the composition of the microbial community.

To compare microbial communities between particulate and filtered water samples, relative abundances of each community were determined at the phylum level. We observed that the bacterial community bound to the particulate matter was dominated by Actinobacteriota at a relative abundance of 33%, while the filtered water was dominated by Proteobacteria at a relative abundance of 47%, and both communities contained Bacteroidota (Fig. 5c). As only nine Actinobacterial genera are known for biofilm formation, while Proteobacteria are commonly found to produce biofilms on SPM, these results are not in line with our expectations based upon current literature [37,38]. Nevertheless, the particle-bound community had fewer total operational taxonomic units (OTUs) present when compared to the bulk river water, and it was dominated by a few phyla notably Actinobacteriota, Bacteroidota, Cyanobacteria and Proteobacteria. While the filtered water was dominated by Proteobacteria and Bacteroidota, many other phyla were present in smaller quantities within the community. Another study, looking at sediment-bound and surface water microbial communities in cascading reservoirs on the Mekong River, found similar trends of communities dominated by Actinobacteria and Proteobacteria but found that Actinobacteria were more abundant within the sediment-bound communities than the surface waters [39]. As colonization of particle surfaces is limited to bacteria capable of forming biofilms, the results agreed with our expectation that the river water contains higher microbial biodiversity than the particle-bound microbiota and that the dominant microbes would differ between the two habitats.

In sampling at the dam, we showed that the mPOT can be deployed in a passive, unattended context and capture and preserve particulate matter from the environment. It also highlighted advantages of this passive sampling system in the ability to monitor SPM accumulation over long periods of time, including the swapping out of the filtration portion to determine the accumulation of SPM over a given period if desired. Long-term installation at sites such as the Moses–Saunders Power Dam or similar installations will allow for additional time-resolved context with which to analyse recovered microplastics and particle-bound communities of microorganisms. By gaining this relevant information, it could indicate sources or seasonal trends of microplastic pollutants and environmental factors and how these drive the formation of the particle-bound microbial communities.

In addition to samples collected by the mPOT, online monitors at the Unit 32 turbine at the dam site allowed for measurements of size-fractionated extracted chlorophyll *a* (Table 2), providing additional context to phototrophic populations within the water collected. While cyanobacteria abundance within the water samples collected was <5%, a higher abundance was noted from particle-bound samples. Future work could seek to collect size-fractionated extracted chlorophyll *a* data from SPM for additional comparisons between the two sample sets.

**Table 2.** Planktonic chlorophyll *a* and taxa abundance present in the St. Lawrence River during mPOT sampling

	Size fractionated extracted chlorophyll-*a*			% abundance of taxa			
**Date**	**Micro (>20 µm**)	**Nano (2–20 µm**)	**Pico (0.2–2 µm**)	**Chlorophyta and Euglenophyta**	**PC-rich Cyanobacteria**	**Heterokontophyta and Pyrrophyta**	**Cryptophyta and PE-rich Cyanobacteria**
8/20/21	0.29	0.77	2.01	21	18	50	11
8/21/21	0.42	0.84	2.08	21	17	50	12

### Active sampling of river water from a riverbank

The objectives of the sampling use case on the Genesee River in Rochester, NY were to characterize any microplastics detected in an urban waterway and to test the procedure for training citizen scientists to deploy mPOT devices. Though no microplastics were recovered, the sampling proved effective in recovering SPM from the river system, and the mPOT proved simple to utilize by individuals with little training or prior knowledge of the device.

**Fig. 6.**
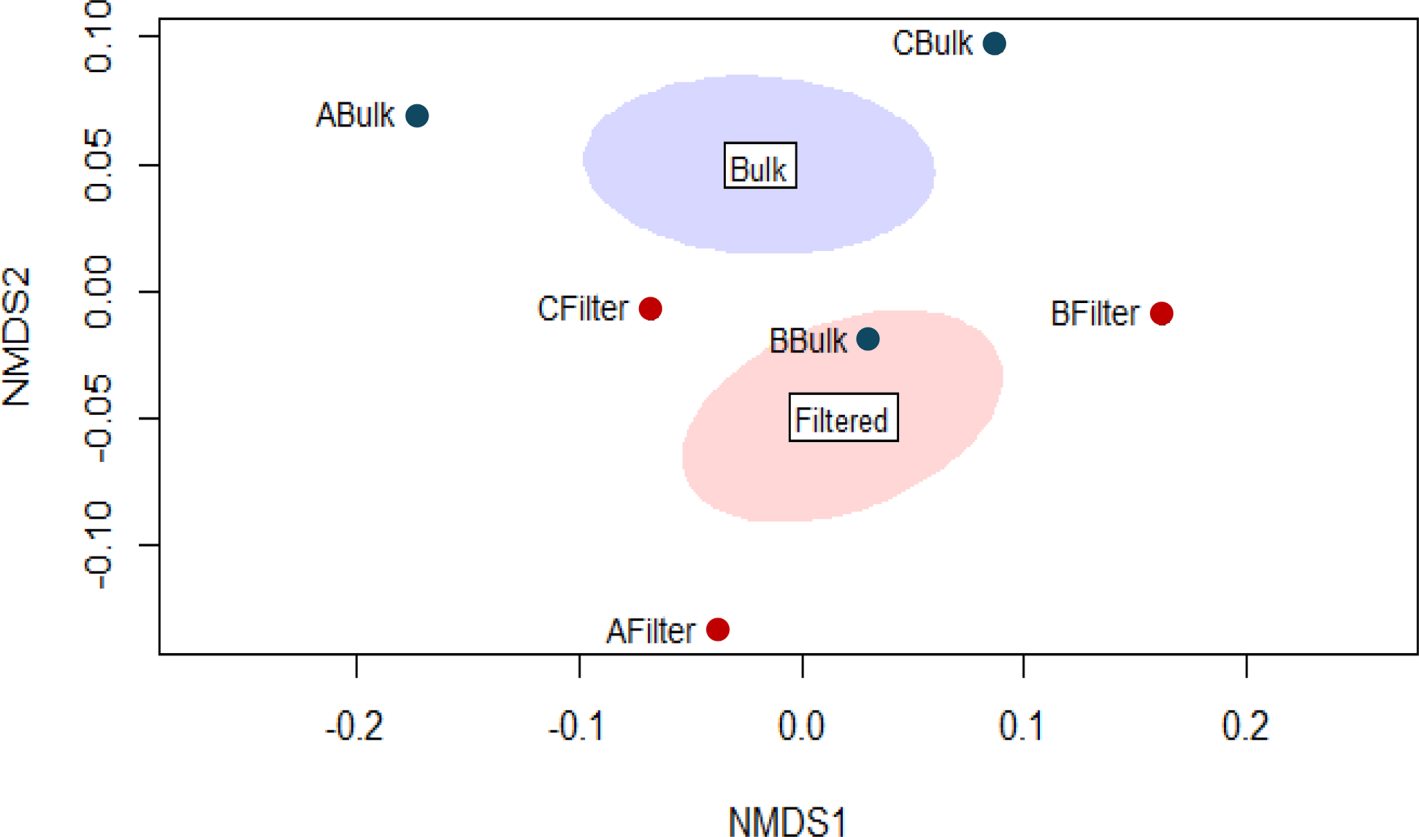
NMDS plot comparing bacterial community composition of surface water and through sampling device filtrate from the Genesee River by beta-diversity metric, UniFrac. Samples are from three sites (A, B and C) and were either total water communities (Bulk) or SPM bound (Filter).

Following 16S rRNA gene amplicon sequencing of the particle-bound and bulk water bacterial communities, ecological diversity indices were calculated for alpha and beta diversity. We generated a non-metric multidimensional scaling (NMDS) plot from a distance matrix of beta diversity index weighted UniFrac values for the microbial communities, which showed distinct community formations when comparing bulk water and filtered particulates (Fig. 6). Higher average alpha diversity was found within the particle-bound community at the three sites when compared to the bulk water community, indicated by higher Shannon diversity indices of 4.81 and 3.86, respectively, a metric accounting for species richness and evenness.

**Fig. 7.**
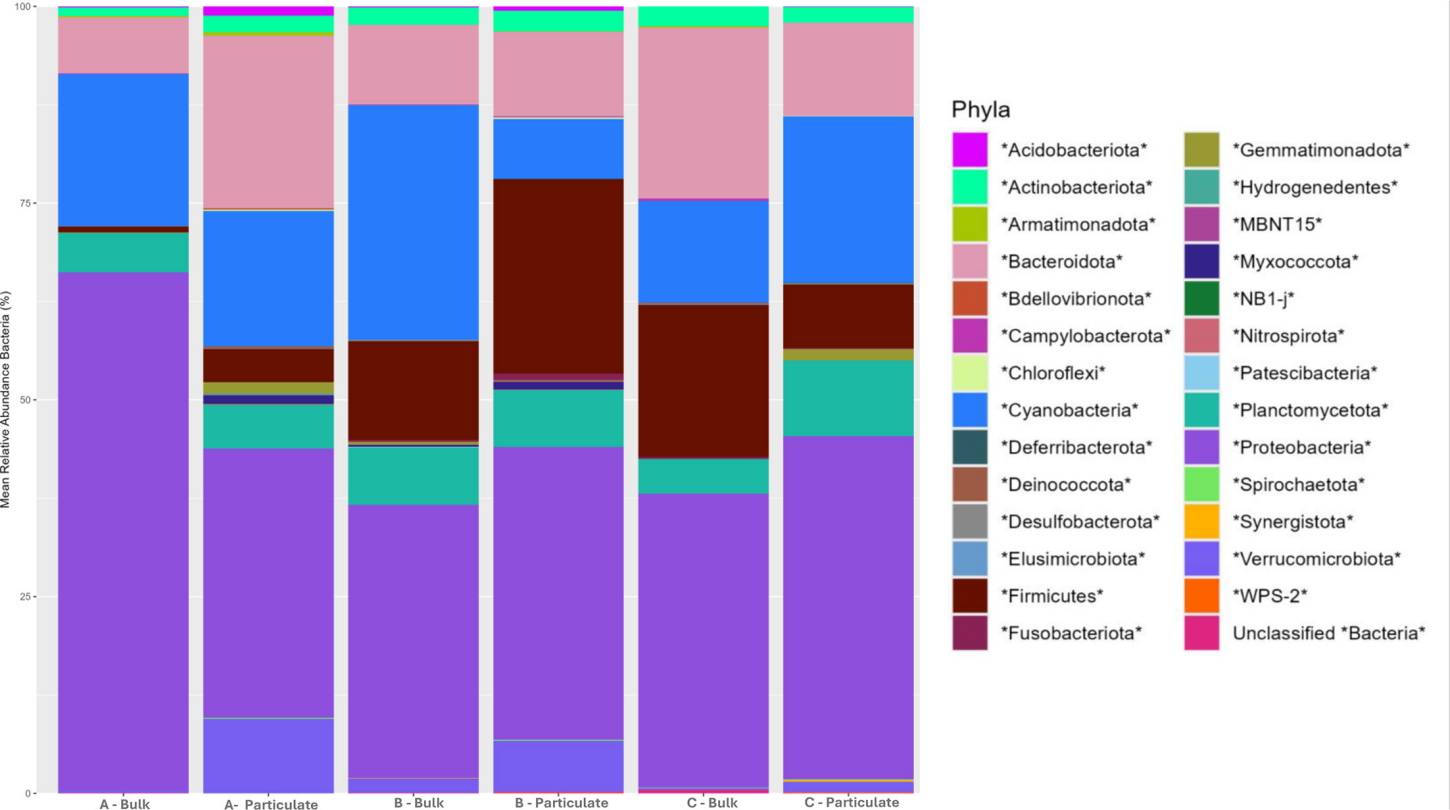
Taxonomic bar plot of bacterial phyla relative abundance of bulk water and particulate matter from the Genesee River. Samples are from three sites (A, B and C) and were either total water communities (Bulk) or SPM bound (Particulates).

As these samples were taken from within a 0.5-mile stretch of the Genesee River, microbial communities across the three sites were similar in composition, but not in relative abundances of some of the members present (Fig. 7). Notably, members of the phyla Verrucomicrobiota were found in relative abundancies less than 1% in bulk water samples from both Site A and C, and at 1.8% in bulk water from site B. In contrast, Verrucomicrobiota comprised 9.4, 6.4 and 1.3% for particle-bound microbial communities from sites A, B and C respectively, which is expected as Verrucomicrobiota has been found to preferentially associate with particulate matter in aquatic systems [40]. Though the relative abundance of the phyla Proteobacteria, the most abundant taxa amongst all samples, was similar among all sites, particle-associated communities had a wider variety of Proteobacteria, with an average of 55 Proteobacterial OTUs represented in bulk water compared to the 90 OTUs represented in the particle-associated. A similar trend was observed with Bacteroidota, with an average of 30 OTUs present for bulk water samples and 61 OTUs for particle-associated samples. The increased diversity amongst the most abundant phyla, in addition to the higher total amount of OTUs present, within the particle-bound communities show a more diverse microbial community forming within the particle-bound community compared to bulk water, in contrast to what was seen at the Moses–Saunders Power Dam. Microbial community formation may have been influenced by temperature, as this was the only sampling with the mPOT during the winter, where water temperatures averaged 2 °C, potentially leading to a more diverse community in particle-bound fraction compared to the bulk water. Additionally, while microplastics are commonly found in concentrations ranging from 5 to 10 items/l in urban freshwater [41], limitations in sampling duration of 1 h or microplastic extraction methodology, specifically in the usage of 1.2 g/cm^3^ sodium chloride salt solution rather than a higher density solution, may have resulted in no microplastics being identified from collected samples.

### Passive sampling from Barker’s Island within the St. Louis River Estuary

As Lake Superior and the St. Louis River Estuary have seen a historical rise in algal bloom occurrences within the last decade, the composition of microbial communities, both in bulk water and bound to SPM, were analysed with a particular interest in cyanobacterial abundance, as well as between the 2 months in late summer, when blooms are most likely to occur. We hypothesized that cyanobacterial abundance would be highest in the particle-bound communities, as blooms tend to form mats along the surface of the water where they would be more likely to interact with SPM floating along the surface.

Using analyses described previously, alpha and beta diversity metrics were calculated for each of the samples collected during the two 1-month-long sampling periods, and NMDS plots were generated from a distance matrix of beta diversity index weighted UniFrac values for the microbial communities. Clustering of samples indicated that the sampling period and sample type (i.e. particle bound vs. planktonic) were the primary drivers of differences between microbial communities, with samples collected during August.

**Fig. 8.**
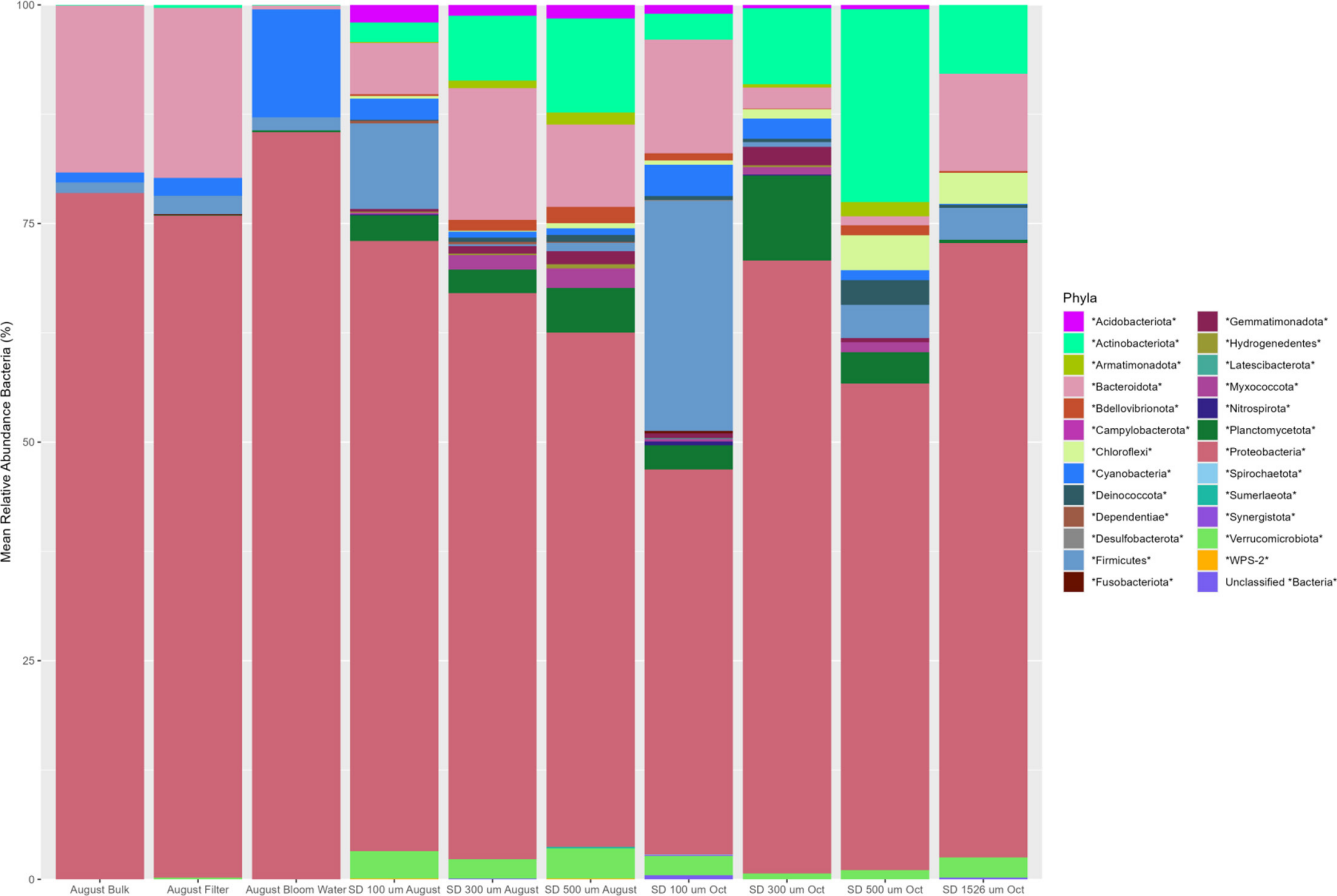
Taxonomic bar plot of bacterial phyla relative abundance of size-fractionated SPM from Barker’s Island. Samples were collected in August and October and were size-fractionated into 100, 300, 500 and 1,526 µm.

Once again, samples from both bulk water and particle-bound communities were dominated by Proteobacteria, with Bacteroidota again being highly prevalent among samples (Fig. 8). Actinobacteriota was noted in all samples, though its relative abundance was much higher in particle-bound communities than in bulk water collected. Size fractionated distinction amongst communities was also found, most notably in the higher relative abundance of *Firmicutes* on the 100 µm particles compared to the 300, 500 and 1,526 µm particles for both sampling events. A similar trend was also noted for Cyanobacteria, where relative abundancies were higher on smaller sized particles compared with both larger particles and bulk water communities, save for the water collected from Barker’s Island Swimming Beach where an algal bloom occurred. Similar results were seen in a study characterization of sediment-associated bacterial communities across the Great Lakes [42], with Proteobacteria, Actinobacteria and Acidobacteria dominating the communities sequenced. This study also found increases in Firmicutes within Lake Superior and Lake Ontario, though there was no analysis of different particle sizes within this study. Previous microplastic studies on Lake Superior have found wide variability in concentrations, ranging from 4,000 to 100,000 particles/km^2^ [43], though sampling commonly occurs on the surface waters, rather than near shore.

Our sampling in the St. Louis River Estuary again highlighted distinct microbial community formation between particle-associated and bulk water samples. While other experiments using mPOT focused on this distinction, as highlighted with diversity metrics (Table 3), this sampling identified changes in microbial communities on SPM based on particle size, finding that the mPOT was suitable for size-fractionated recovery of SPM and the subsequent analysis of microbial communities formed on these particles.

**Table 3.** Microbial diversity indices from samples collected using mPOT

Samples			Diversity indices				
**Location**	**Type**	**Date(s) collected**	**Berger-Parker**	**Chao1**	**Shannon**	**Simpson**	**Ace**
Moses–Saunders Power Dam	Bulk water	8/20/21	0.04	3751.35	5.65	0.01	4700.60
	Particulate - all sizes	8/20/21	0.15	996.25	4.19	0.05	1130.66
Genesee River	Bulk water: Site A	1/14/23	0.24	363.87	3.08	0.10	570.20
	Particulate; Site A, all sizes	1/14/23	0.06	437.33	4.91	0.01	422.18
	Bulk water - Site B	1/14/23	0.14	512.44	4.20	0.04	661.94
	Particulate: Site B, all sizes	1/14/23	0.05	1151.00	5.24	0.01	2206.26
	Bulk water: Site C	1/14/23	0.08	362.78	4.29	0.03	353.09
	Particulate: Site C, all sizes	1/14/23	0.09	614.05	4.29	0.03	764.02
Barker’s Island	Bulk water	8/31/23	0.19	143.50	2.38	0.13	174.94
	Filtered water	8/31/23	0.31	146.11	2.39	0.17	233.94
	Bulk water: Algal bloom	8/31/23	0.34	113.00	2.31	0.17	186.87
	Particulate: 100 µm	7/17/23 to 8/31/23	0.40	628.95	3.58	0.17	994.33
	Particulate: 300 µm	7/17/23 to 8/31/23	0.12	465.90	4.30	0.04	685.93
	Particulate: 500 µm	7/17/23 to 8/31/23	0.13	619.80	4.69	0.03	945.32
	Particulate: 100 µm	9/1/23 to 10/17/23	0.14	559.51	4.87	0.03	746.26
	Particulate: 300 µm	9/1/23 to 10/17/23	0.27	574.70	4.09	0.08	773.59
	Particulate: 500 µm	9/1/23 to 10/17/23	0.16	404.52	4.44	0.04	452.30
	Particulate: 1,526 µm	9/1/23 to 10/17/23	0.15	266.50	3.95	0.04	378.40

## Conclusion

We have developed a new SPM sampling device, easily accessible and affordable for researchers and citizen scientists for usage in varying aquatic systems. The mPOT was effective in recovery of microparticles down to –100 µm in size during lab validation and was able to recover similarly sized SPM from a variety of freshwater systems while deployed at sites unsuitable for trawling nets. The mPOT separated particulate matter by size and enabled analysis of particle-bound microbial communities across various freshwater environments.

The simple design and relatively inexpensive construction cost of this sampling device facilitate operation to enable citizen scientists to easily utilize mPOT with their local communities in their local waterways, though samples must be analysed within laboratories with proper instrumentation to quantify microplastics and identify microbial populations.

While commonly employed SPM samplers have an advantage in total volumes of water able to be filtered in a short period of time, the mPOT allows for better analysis of suspended particulates within specified compartments of aquatic systems. Pumping water into the sampling device allows for sampling of suspended particles at varying depths of a water column, or for sampling at previously difficult to reach locations.

We have demonstrated advantages in passive sampling with interchangeable filters, allowing for determination of plastic accumulation over a given period of time and size-fractionated recovery, modifiable with changing of mesh sizes used. The mPOT is therefore best suited for inexpensive and consistent recovery of suspended microparticles from systems not easily sampled by other sampling methods, including groundwater systems and for recovery of particles smaller than 300 µm in size. Limitations in plastic recovery likely stem from the total quantity of water sampled, as microplastics are often widely dispersed across these bodies of water. To address this, long-term passive sampling is recommended for future studies using mPOT which seeks to target microplastic recovery.

While we hypothesized that bulk water communities would tend to be more diverse than particle-bound communities, our results showed that this was not the case in either the Genesee River or Barker’s Island within the St. Louis River Estuary. Similar studies have found that plastic-bound microbial communities tend to be less species rich on average when compared to surrounding seawater, though plastic-bound communities were less likely to be dominated by the most abundant organisms found within the sample. Future studies could identify under which conditions these microbial communities form and characterize interactions between members of these communities which drive their composition.
